# Facilitators and Barriers to Adopting Robotic-Assisted Surgery: Contextualizing the Unified Theory of Acceptance and Use of Technology

**DOI:** 10.1371/journal.pone.0016395

**Published:** 2011-01-20

**Authors:** Christine BenMessaoud, Hadi Kharrazi, Karl F. MacDorman

**Affiliations:** 1 Indiana University School of Informatics, Indianapolis, Indiana, United States of America; 2 Purdue University School of Engineering and Technology, Indianapolis, Indiana, United States of America; University of Oxford, United Kingdom

## Abstract

Robotic-assisted surgical techniques are not yet well established among surgeon practice groups beyond a few surgical subspecialties. To help identify the facilitators and barriers to their adoption, this belief-elicitation study contextualized and supplemented constructs of the unified theory of acceptance and use of technology (UTAUT) in robotic-assisted surgery. Semi-structured individual interviews were conducted with 21 surgeons comprising two groups: users and nonusers. The main facilitators to adoption were Perceived Usefulness and Facilitating Conditions among both users and nonusers, followed by Attitude Toward Using Technology among users and Extrinsic Motivation among nonusers. The three main barriers to adoption for both users and nonusers were Perceived Ease of Use and Complexity, Perceived Usefulness, and Perceived Behavioral Control. This study's findings can assist surgeons, hospital and medical school administrators, and other policy makers on the proper adoption of robotic-assisted surgery and can guide future research on the development of theories and framing of hypotheses.

## Introduction

Technological innovations have spawned the development of new surgical techniques. For certain operations, open surgery has given way to standard laparoscopic and robotic-assisted surgery, in which surgeons use micro equipment through small incisions [1], [2], [3]. Nevertheless, many surgeons resist incorporating robotic-assisted surgery into their routine practice. The purpose of this qualitative study is to understand the rationale behind surgeons' decision to reject or adopt robotic-assisted surgical techniques.

This study attempts to elicit common beliefs among surgeons to contextualize the unified theory of acceptance and use of technology (UTAUT) in robotic-assisted surgery. The elicited beliefs, obtained from in-depth interviews, are used to identify dominant UTAUT constructs. Consequently, this study attempts to answer two research questions: 1) What are the most important facilitators and 2) barriers to surgeon's adoption of robotic-assisted surgery?

So far, little research has been conducted on this topic and, as with other health technologies, the application of theory to the study of physicians' behavior has been limited [4]. This is the first study to apply a technology acceptance model to surgeons' adoption of surgical robots. Based on data from the study, the UTAUT model is modified to include two new main constructs, Attitude toward Using Technology and Leadership. This study also identifies the most important facilitators and barriers to the adoption of robotic-assisted surgery.

### Robotic-Assisted Surgery

The following paragraphs briefly review the advantages and disadvantages of open, laparoscopic, and robot-assisted surgery (Table 1):

**Table 1:** Advantages and disadvantages of various surgical techniques.

Type of Surgery	Advantages	Disadvantages
Open	Fully exposed surgical siteDirect access to structures and organsAffordablePervasiveWidest variety of operationsFor morbidly obese patientsFor patients with prior operationsFor patients with multiple adhesions	Large incisionPainful recoveryLengthy healing processProlonged hospital staysHigher infection ratesLarge scar and disfigurement
Laparoscopic	AffordablePervasiveShorter hospital staysReduced postoperative painLower incidence of wound infectionsGreatly enhanced cosmetic outcomesImproved patient outcomes	Limited dexterityLoss of depth perceptionRisk of camera instabilityRisk of port placementAwkward movement of instrumentsFulcrum effectPoor ergonomicsFatigueReversed image can cause miscommunicationNot for morbidly obese patients
Robotic-Assisted	Greater dexterityElimination of hand tremorElimination of fulcrum effectEnhanced depth perceptionCamera stabilityImproved surgeon ergonomicsIncreased accuracyScalable motionsPotential for micro-anastomosesPotential for telesurgeryShorter hospital staysReduced postoperative painLower incidence of wound infectionsGreatly enhanced cosmetic outcomesImproved patient outcomes	High cost (purchase, upgrade, maintenance)Long surgeon training timeLong set-up timeBulkiness of equipmentLack of tactile and force feedbackRisk of malfunction or failureRisk of port placementPatient safety during emergencyRequires additional staff trainingNot for morbidly obese patients

#### Open Surgery

Open surgery consists of cutting skin and tissues to expose organs and other structures. Open surgery provides direct access to the operative site with depth perception and dexterity for one or more sets of hands. Open surgery is the only option for many procedures and for certain kinds of patients (e.g., obese patients, patients with prior surgeries or multiple adhesions). However, open surgery usually requires a long incision, which leaves a visible scar. The trauma caused in gaining access to the organs can result in a more painful recovery, a longer healing process, prolonged hospital stays, a higher risk of infection [5], [6], and sometimes even disability and morbidity [7].

#### Laparoscopic Surgery

Minimally invasive procedures have advantages for certain kind of operations [6], [8]: shorter hospital stays, reduced postoperative pain, fewer infections, and better cosmetic outcomes [3]; however, they also have disadvantages for the surgeon: limited dexterity, loss of depth perception, camera instability, awkward movement of instruments and scopes (e.g., fulcrum effect), poor ergonomics, fatigue, and miscommunication caused by the reversed image on the monitor [5], [6].

#### Robotic-Assisted Surgery

A surgical robot is a self-powered, computer-controlled manipulator that can be programmed to aid in positioning and manipulating surgical instruments [9]. The robotic manipulator acts as a remote arm extension governed by the surgeon's movements [3], [10]. Robotic-assisted surgical techniques can enable surgeons to carry out more complex tasks than standard laparoscopic surgery and achieve better performance for specific operations [11]. Other advantages include greater dexterity and accuracy, scalable motions, camera stability, improved surgeon ergonomics, elimination of tremor, depth perception, and better patient outcomes [5], [6], [10], [12], [13]. Surgical robots also help hospitals market themselves as cutting edge [11]. However, a robotic system lacks tactile and force feedback [2], [9], affords the surgeon less control over patient safety [14], has the risk of malfunction or failure [14], has risks associated with port placement [15], is bulky, suffers incompatibilities with conventional laparoscopic instruments, has less availability of parts [13], and sometimes requires surgeon troubleshooting [14]. A further disadvantage is that a robotic procedure can take longer than a standard laparoscopic procedure because of increased setup time [16]. Robotic-assisted surgery also costs more than other techniques because of the fixed cost of the robotic system (on average $1.5 million [17], higher maintenance and support costs [12], and the cost of expensive equipment upgrades. Nevertheless, robotic-assisted procedures receive the same reimbursement in the United States as laparoscopic procedures from commercial health insurance and federally administered Medicare [18].

This study focuses on the da Vinci surgical system because of its extensive use in diverse subspecialties [13], [19], [20], [21], [22], [23]. The da Vinci is manufactured by Intuitive Surgical, Inc. Currently, the da Vinci is the only actively marketed surgical system to have received approval from the US Food and Drug Administration and the only system to have received approval for cutting and suturing. The da Vinci consists of a surgeon's console, four robotic arms, and a video tower [6].

The use of robotic systems has been gaining popularity in several surgical subspecialties, such as cardiac, thoracic, urological, gynecological, pediatric, and general surgery [24]. However, despite this growth, some surgeons in these specialties are not inclined to adopt robotic-assisted surgery. Healthcare professionals, and especially physicians, can be slow in adopting new technologies [25]. Common factors in slow adoption have been incorporated in the main constructs and subconstructs of the UTAUT model [26]. Some of these factors have been identified as important to the acceptance of healthcare technology. However, the UTAUT model has not been specifically applied to robotic-assisted surgery. In doing so, the following factors proposed by previous research are relevant:

#### Self-Governance

Physicians' adoption of a technology is highly self-governed because of the specialized services they provide [27]. Thus, physicians' perception of a technology is the most important factor in its voluntary use.

#### Long Training Requirements

Lack of acceptance may be attributed to the challenges of coping with new devices, adapting new kinds of instrumentation, and learning new operative maneuvers [28]. As a result, surgeons have to interrupt their practice to attend educational programs, seminars, and training sessions. For some devices like surgical robots, uniform training standards among hospitals are lacking.

#### Lack of Clear Benefits

Some surgeons believe traditional practices are sufficient to treat their patients. For example, previous research has found that in general surgery the application of robotic technology has not translated into improved patient outcomes [16].

### Unified Theory of Acceptance and Use of Technology (UTAUT)

Research on robotic-assisted surgery tends to focus on the development and implementation of the robotic technology and the evaluation of clinical outcomes [29] without sufficiently considering how readily surgeons would accept the technology. An individual's intentional or voluntary use of a technology is referred to as technology acceptance [30], [31]. Technology acceptance models originate from the theory of reasoned action [32], [33], a general social-psychological/behavioral theory. To adapt the theory of reasoned action to information technology use various studies were conducted to determine which variables to include [30], [31]. Several models were proposed, such as the technology acceptance model (TAM) [31], and a version of TAM including social influences [34]. Recent efforts to unify the technology acceptance literature resulted in the unified theory of acceptance and use of technology [26].

**Figure 1:**
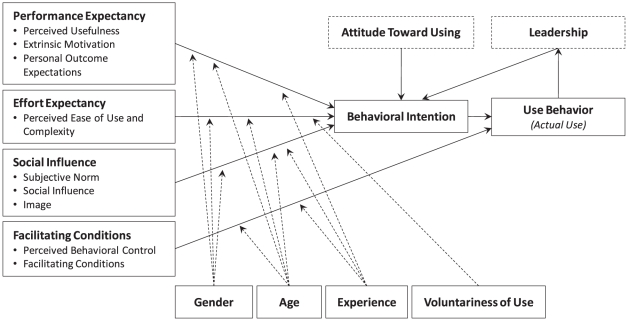
Modified Unified Theory of Acceptance and Use of Technology (UTAUT) model contextualized for robotic surgery.

To provide a better understanding of the facilitators and barriers to surgeons' adoption of robotic-assisted surgery, this research uses the UTAUT model (Figure 1). The UTAUT model consists of four main constructs that directly determine user acceptance and usage behavior: Performance Expectancy, Effort Expectancy, Social Influence, and Facilitating Conditions [26]. Performance Expectancy is defined as the degree to which an individual believes that using the system will improve job performance. Effort Expectancy is the degree to which an individual believes the system is easy to use. Social Influence is the degree to which an individual believes that important others think he or she should use the system. Facilitating Conditions is the degree to which an individual believes that an organizational and technical infrastructure exists to support the use of the system. (Venkatesh et al. [26] refer to main constructs as key constructs and the subconstructs of the main constructs as root constructs. Note that Facilitating Condition, with a broader definition, is a main construct and should not be confused with the similar name of its subconstruct, which is more narrowly defined.)

To mediate the impact of the four main constructs on usage intention and behavior, four factors are posited: gender, age, experience, and voluntariness of use. The UTAUT model was developed through a review and consolidation of the constructs of eight models that earlier research had employed to explain information technology usage behavior. Validation of the UTAUT model in a longitudinal study found it accounted for about 70% of the variance in behavioral intention (i.e., intention to adopt a new technology) and about 50% of the variation is actual use of the technology [26].

Because the UTAUT model has not yet been applied to the adoption of surgical robots, it requires contextualization. Previous research has proposed belief elicitation as the preferred method for contextualizing theories of behavior in a specific setting (e.g., healthcare), with a new population (e.g., surgeons), and a new behavior of interest (e.g., robotic-assisted surgery) [35]. Indeed, this elicitation study was aimed at discovering surgeons' beliefs based on their responses to open-ended questions investigating the positive and negative influence of UTAUT constructs. Consequently, these beliefs helped not only to contextualize the UTAUT model for robotic-assisted surgery but also to extend the model.

## Results

**Figure 2:**
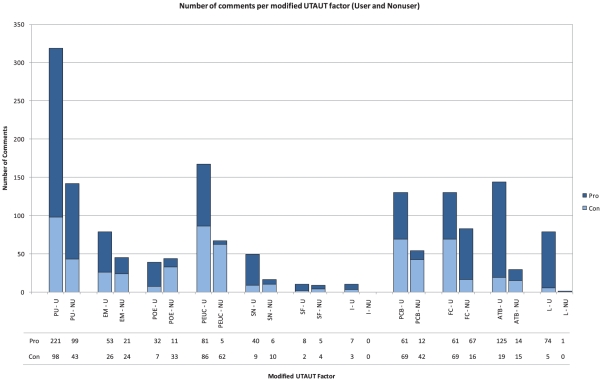
Number of comments (pro-robotic surgery versus contra-robotic surgery) per modified UTAUT subconstruct for users and nonusers. Note: PU – Perceived Usefulness, EM – Extrinsic Motivation, POE – Personal Outcome Expectations, PEUC – Perceived Ease of Use/Complexity, SN – Subjective Norm, SF – Social Factor, I – Image, PBC – Perceived Behavioral Control, FC – Facilitating Conditions, ATU – Attitude Toward Using Technology, L – Leadership.

The results of the content analysis were organized using the modified UTAUT model (Figure 1). The questionnaire (Appendix S1) elicited comments in all six main constructs: Performance Expectancy, Effort Expectancy, Attitude Toward Using Technology, Social Influence, Facilitating Conditions, and Leadership. Table 2 and Figure 2 summarize the categorization of comments from both robot users and nonusers regarding each modified UTAUT construct.

**Table 2:** The comments of users and nonusers concerning robotic surgery are categorized according to the modified UTAUT constructs.

Main construct	Subconstruct	Users' Comments			Nonusers' Comments		
		Pro	Neutral	Con	Pro	Neutral	Con
Performance Expectancy	Perceived Usefulness	221 (60%)	51 (14%)	98 (26%)	99 (62%)	18 (11%)	43 (27%)
	Extrinsic Motivation	53 (40%)	54 (41%)	26 (19%)	21 (30%)	24 (35%)	24 (35%)
	Personal Outcome Expectation	32 (68%)	8 (17%)	7 (15%)	11 (17%)	22 (33%)	33 (50%)
Effort Expectancy	Perceived Ease of Use & Complexity	81 (47%)	5 (3%)	86 (50%)	5 (7%)	0 (0%)	62 (93%)
Social Influence	Subjective Norm	40 (82%)	0 (0%)	9 (18%)	6 (22%)	11 (41%)	10 (37%)
	Social Factors	8 (80%)	0 (0%)	2 (20%)	5 (38%)	4 (31%)	4 (31%)
	Image	7 (64%)	1 (9%)	3 (27%)	0 (0%)	0 (0%)	0 (0%)
Facilitating Conditions	Perceived Behavioral Control	61 (36%)	40(23%)	69 (41%)	12 (22%)	1 (2%)	42 (76%)
	Facilitating Conditions	208 (84%)	24 (10%)	16 (6%)	67 (81%)	0 (0%)	16 (19%)
Attitude Toward Using Technology		125 (68%)	40 (22%)	19 (10%)	14 (32%)	15 (34%)	15 (34%)
Leadership		74 (94%)	0 (0%)	5 (6%)	1 (100%)	0 (0%)	0 (0%)

Percentages are calculated across each subconstruct separately for users and nonusers.

The most discussed main constructs were Performance Expectancy (users: 550 comments, 37% of all user comments; nonusers: 295 comments, 50% of all nonuser comments) and Facilitating Conditions (users: 418, 50%; nonusers: 138, 23%). These two constructs had the highest percentage of pro-adoption and contra-adoption comments among users and nonusers (Table 3). The most discussed subconstructs were Perceived Usefulness (users: 370, 25%, nonusers: 160, 27%) and Facilitating Conditions (users: 248, 17%, nonusers: 83, 14%). Social Factors and Image were the least discussed subconstructs.

**Table 3:** Proportion of comments categorized according to the modified UTAUT main constructs.

Main construct	Users' Comments			Nonusers' Comments		
	All	Pro	Con	All	Pro	Con
Performance Expectancy	037%	034%	039%	050%	055%	040%
Effort Expectancy	012%	009%	025%	011%	002%	025%
Facilitating Conditions	029%	030%	025%	023%	033%	023%
Attitude Toward Using Technology	012%	014%	006%	008%	006%	006%
Social Influence	005%	006%	005%	007%	004%	006%
Leadership	005%	008%	001%	000%	000%	000%
**Total**	100%	100%	100%	100%	100%	100%

Percentages are calculated across all main constructs. All: Percentage of total comments; Pro: Percentage of pro-adoption comments; Con: Percentage of contra-adoption comments.

The comments were separated into facilitators and barriers to adoption. Among the subconstructs, the major facilitators for adoption were Perceived Usefulness (users: 221, 24%, nonusers: 99, 41%) and Facilitating Conditions (users: 208, 23%, nonusers: 67, 28%). The next main facilitators were Attitude Toward Using Technology among users (125, 14%) and Extrinsic Motivation among nonusers (21, 9%). The main barriers to adoption were Perceived Ease of Use and Complexity (users: 86, 29%, nonusers: 62, 17%), Perceived Usefulness (users: 98, 25%, nonusers: 43, 25%), and Perceived Behavioral Control (users: 69, 20%, nonusers: 42, 17%).

## Discussion

This study was conducted to find the most important facilitators and barriers to surgeons' adoption of robotic-assisted surgery. To this end, the discussion of the results is divided into six subsections: facilitators, barriers, a comparison of users and nonusers, sample comments, limitations, and contextualized solutions to address the adoption issues.

### Facilitators

#### Users

In relation to the proposed modified UTAUT model, Perceived Usefulness, Facilitating Conditions, and Attitude toward Using Technology were the key facilitators of users' adoption of robotic-assisted surgery (Table 4).

**Table 4:** Proportion of comments categorized according to the modified UTAUT subconstructs.

Subconstruct	Users' Comments			Nonusers' Comments		
	All	Pro	Con	All	Pro	Con
Perceived Usefulness	025%	**024%**	**029%**	027%	**041%**	**017%**
Facilitating Conditions	017%	**023%**	005%	014%	**028%**	006%
Perceived Ease of Use & Complexity	012%	009%	**025%**	011%	002%	**025%**
Extrinsic Motivation	009%	006%	008%	012%	009%	**010%**
Perceived Behavioral Control	012%	007%	**020%**	009%	005%	**017%**
Attitude Toward Using Technology	012%	**014%**	006%	008%	006%	006%
Personal Outcome Expectation	003%	004%	002%	011%	005%	**013%**
Subjective Norm	003%	004%	003%	005%	002%	004%
Leadership	005%	008%	001%	000%	000%	000%
Social Factors	001%	001%	001%	002%	002%	002%
Image	001%	001%	001%	000%	000%	000%
**Total**	100%	100%	100%	100%	100%	100%

Percentages are calculated across all subconstructs. The main facilitators and barriers to robotic-surgery adoption among users and nonusers are indicated in bold (10% cutoff point). Values are sorted based on average total percentage among users and nonusers (not shown). All: Percentage of total comments; Pro: Percentage of pro-adoption comments; Con: Percentage of contra-adoption comments.

Within the Performance Expectancy main construct, the most influential subconstruct (with 221 pro-adoption comments) was Perceived Usefulness. Users were attracted to the many enhanced functions of the robot: better visualization, increased precision, better dexterity, elimination of hand tremor, better suturing, better instrumentation, better angle of placement, easier access, and better ergonomics. Another persuasive element in this construct is that robotic technology improves on standard laparoscopy, allowing the performance of closed surgeries as if they were open. In addition, the users trusted this technology because of the reliability of the robot. In the Extrinsic Motivation subconstruct, improved patient outcomes influenced the surgeons' decision to adopt surgical robots. These positive outcomes include reductions in bleeding, pain, blood clots, infection rate, complication rate, and post-operative adhesions. Personal Outcome Expectations indicated that robotic-assisted surgeons had experienced an increase in patient referrals, which in turn increased job satisfaction.

Within the Social Influence main construct, the most influential subconstruct with 40 pro-adoption comments was Subjective Norm. Surgeons viewed robotic technology as market driven. Because of public advertisement, media coverage, and online information, patients are encouraged to seek out the latest technology. These patients are thus attracted to hospitals and surgeons where robotic-assisted surgery is available and marketed. In this study, the users claimed not to care about the effects of performing robotic-assisted surgery on their personal image; users stated that they performed robotic-assisted surgery, because they wanted to improve patient outcomes.

Within the Facilitating Conditions main construct, the most influential subconstruct (with 208 pro-adoption comments) was Facilitating Conditions. Surgeons expressed confidence in using this technology because of the support provided by Intuitive Surgical, Inc. A technical support representative is onsite most of the time or easily reached by phone.

Attitude Toward Using Technology is another main construct that had a definite impact on the sample's adoption of robotic technology. The construct contains 125 pro-adoption comments made by the robot users. Most users indicated that they have fun performing robotic-assisted surgery. They held high expectations for the future development of this technology, such as the ability to perform surgery remotely and the improved portability of the robot. Development of additional practice-specific instruments will allow the adoption of this technology by other specialties, such as general surgery, cardiac surgery, and plastic surgery. Surgeons also indicated that as they became more experienced using the technology, their technique and comfort level improved. Overall, these surgeons are early adopters [1]; their positive attitude toward using new technology has a great impact on their behavior.

The Leadership subconstruct was added to contextualize the UTAUT model for this study. Most robot users are involved in training and proctoring other surgeons and otherwise helping them to adopt this technology. Some of these surgeons also help the manufacturers make instruments more effective and durable. These improvements allow the specialties to accommodate more procedures.

#### Nonusers

In relation to the modified UTAUT model, similar to the users, the results among the nonusers showed that Perceived Usefulness and Facilitating Conditions are the subconstructs that increase surgeons' expected success in adopting robotic-assisted surgery (Table 4).

Within the Performance Expectancy main construct, the most influential subconstruct with 99 pro-adoption comments was Perceived Usefulness. Persuasive elements in this construct were similar to those identified by robot users. The Social Influence main construct has a slightly positive influence on robotic-assisted surgery adoption with 10 pro-adoption comments. Robot nonusers expressed sentiments similar to those of users: that the technology is market-driven. Within the Facilitating Conditions main construct, the Facilitating Conditions subconstruct has 67 pro-adoption comments. Even robot nonusers aware of the good onsite technical support. Training provided by the technical support team was also identified as a positive influence on surgeons' likelihood of adopting robotic technology in the future.

### Barriers

#### Users

In relation to the modified UTAUT model, the results among the users showed that Perceived Usefulness, Perceived Ease of Use and Complexity, and Perceived Behavioral Control were the major barriers to the adoption of robotic-assisted surgery (Table 4).

Within the Effort Expectancy main construct, Perceived Ease of Use and Complexity, the only subconstruct, received 86 contra-adoption comments. Among the contra-adoption comments, a steep learning curve was a major barrier. Surgeons were accustomed to feeling the organs, including the amount of pressure being applied to an organ, during an operation. However, that tactile feedback is lost with robotic technology. Surgeons stated they needed to perform at least 25 operations to learn to ‘feel’ with their eyes. Another element that slowed the decision to adopt robotic technology is the setup of the robot, which is cumbersome and time-consuming.

#### Nonusers

The results among the nonusers showed that Perceived Usefulness, Personal Outcome Expectation, Perceived Ease of Use and Complexity, and Perceived Behavioral Control were the four major barriers to the adoption of robotic-assisted surgery (Table 4).

Like the users, within the Effort Expectancy main construct, Perceived Ease of Use and Complexity was the best represented subconstruct with 62 contra-adoption comments. Among the contra-adoption comments, a steep learning curve was again a major barrier. A lack of tactile feedback is the main reason. Unlike robot users, nonusers did not want to take the time to learn to ‘feel’ with their eyes, and they did not want to learn on their patients. Another barrier was increased operative time because of the time required to set up the robot.

Performance Expectancy is another main construct that has a negative impact on the adoption of robotic technology among nonusers. Among its subconstructs, Personal Outcome Expectation contained 33 contra-adoption comments, and Extrinsic Motivation contained 24 contra-adoption comments. Some nonusers stated that robotic-assisted surgery is merely a marketing tool. In OB/GYN and urology, some robot nonusers said they were losing patients to surgeons who perform robotic-assisted surgery. In cardiothoracic surgery, some surgeons could not justify using a robot because of limited patient volume for the procedures in which a robot can be used.

Despite the advantage of improved post-operative patient outcomes (Table 1), some nonusers noted an increased chance of bowel injury using robotic technology. They argued that the claim that robotic-assisted surgery provides both better margin data and better cancer treatment is false.

Within the Facilitating Conditions main construct, Perceived Behavioral Control had 42 contra-adoption comments. Nonusers noted that hospitals had not encouraged them to perform surgery with the robot. The perceived return on investment was low: Although the robot is an expensive piece of equipment, reimbursement from private insurance companies and Medicare is the same as for regular laparoscopic procedures. Therefore, because the potential for economic gain seemed marginal, surgeons did not want to invest time in learning to use the robot.

### Comparison of Users and Nonusers

Users expressed concerns about the same problems anticipated by nonusers, thus confirming the validity of nonusers' concerns. For example, Perceived Usefulness has been often cited both as a facilitator and a barrier to adoption among both users and nonusers. Both Perceived Ease of Use and Complexity and Perceived Behavioral Control were mostly cited as a barrier, whereas Facilitating Conditions was mostly cited as a facilitator in both groups. Thus, addressing the concerns of users and nonusers may help both groups.

### Sample Comments

Surgeons' comments provide additional insights into the facilitators and barriers to the adoption of robotic-assisted surgery. For example, the following comment reveals concerns that should be addressed by hospital administrators, medical schools, and policy makers:

What scares me to death is that right now the urologic trainees in the US are going to be the most poorly trained urologists surgically that have ever come out of our training program… because surgeries have become minimally invasive, either laparoscopically or robotically. So if the surgeon gets into trouble, nobody in the whole ward knows how to slice the patient open and stop the bleeding to control the problem.

To represent some of these concerns, a selection of common comments is included in Table 5 and Table 6.

**Table 5:** Sample comments made by users based on the modified UTAUT model

Subconstruct	Users	
Perceived Usefulness	Pro	There is a real advantage to using the robot.The benefits of robotic surgery are sharper vision.Robotic surgery is ideally used for very complex laparoscopic surgery.He just realized how truly precisely the robot reproduced his motions.
	Con	There is no difference between the robot and the laparoscopic approach.You lose the tactile sensation of performing surgery.A surgeon must visually feel when too much tension is placed on the tissue.It is difficult to find common operations that can be performed with the robot.
Extrinsic Motivation	Pro	Robot surgery is faster and safer for the patient.The benefit of robotic surgery for the patient is smaller incisions.
	Con	Patient outcomes are similar with the robot or with open surgery.There are certain conditions that exclude a patient from having robotic surgery.
Personal Outcome Expectation	Pro	Because I perform robotic surgery, more physicians refer their patients to me.
	Con	There are no personal benefits to me performing surgery robotically.
Perceived Ease of Use & Complexity	Pro	I find using the da Vinci is very easy.I feel very comfortable with performing robotic surgery.After 25 cases, I could feel the tissue visually, even without tactile feedback.It is easier to learn to use da Vinci from open surgery than to learn general laparoscopy from open surgery.
	Con	It takes time to learn to use the eyes to compensate for the limits of strength.New technology [such as the da Vinci] is a challenge like anything else.There is still an incredible learning curve that the surgeon must try to surmount.Robotic surgery does not save me time.
Subjective Norm	Pro	The reason the hospital purchases the robot is because medicine is competitive.
	Con	The surgeons who don't like to operate [with the da Vinci] want to stick with the techniques they have already mastered.
Social Factors	Pro	Some surgeons perform surgery robotically because robotic surgery is popular.
	Con	The patient is unfamiliar with the procedure performed with robotic technology.
Image	Pro	Robotic surgery simply raises the stature and gives us credibility.
	Con	Surgeons don't want to be taught by peers.
Perceived Behavioral Control	Pro	We need to make sure the younger surgeons are using the robot.Surgeons are the ones who drove the hospital to buy the robot.Surgeons will be attracted to hospitals that have the best technology.
	Con	The large size of the robot is a disadvantage.The robotic system is not simple.It is an expensive piece of equipment.Surgeons do not receive any incentives to use the robot from the hospital.
Facilitating Conditions	Pro	The training was sufficient for a surgeon like me.The technical backup is superb.
	Con	Training in robotic surgery is expensive.I have to close my clinic and take half a day off to get training.
Attitude Toward Using Technology	Pro	Robot surgery becomes exciting… We are light years ahead of what we were thinking.It is as fun as pilots find flying airplanes or race car drivers find racing cars.I love performing surgeries robotically.I like to keep an open mind about technology.
Leadership	Pro	I encourage surgeons to use the robotI have trained more than 30 surgeons in IndianapolisI have been involved in helping to develop new instruments

**Table 6:** Sample comments made by nonusers based on the modified UTAUT model.

Subconstruct	Nonusers	
Perceived Usefulness	Pro	There is definitely a place for robotic surgery.Some of the advantages of the robot are the articulation and the range of motion.The visualization and depth perception are much better than the alternatives.The robot permits the surgeon to perform fine dissection and suturing.The surgeon cannot quite do what the robot can do.
	Con	The fact that robotic surgeons cannot feel is a huge disadvantage.There are enough cases that require me to perform open surgery.
Extrinsic Motivation	Pro	I want the patient to have a good outcome.I recommend it to the majority of patients [whom] are good candidates.
	Con	Being able to see better [does not] correlate to better margin data.
Personal Outcome Expectation	Pro	My practice could advertise and market the robot to try to get more patients.
	Con	I would not be comfortable sacrificing patient outcomes to be able to say I've done something innovative.
Perceived Ease of Use & Complexity	Pro	I think the da Vinci system has made the surgery easier.
	Con	Robotic surgery has a long learning curve.Robotic surgery has a 50 to 100 case learning curve.The disadvantages of robotic surgery are time related.
Subjective Norm	Pro	The pressures to switch to robotic surgery are mainly market driven.Because robotic surgery is new, there is the perception that it may be better.
Social Factors	Pro	Physicians will refer their patient to surgeons who perform robotic surgery.
Perceived Behavioral Control	Pro	The hospital would like to see the robot used.
	Con	There is no reimbursement benefit.The cost of the robot is a disadvantage.Simple procedures can be performed at less cost without the robot.
Facilitating Conditions	Con	I am going to do 50 simulations before performing an actual case.I don't want my patient to be on the table for eight hours for the first case.
Attitude Toward Using Technology	Pro	I am impressed by the science and technology that to into the robot.The role robotics will play in surgery is being worked out.
	Con	I have not taken up robotic surgery because of fear of the unknown.I did not feel comfortable learning on my patients.

Image and Leadership are omitted, because there were no comments.

### Limitations

Because the focus of this research is on the sample's acceptance or rejection of robotic-assisted surgery, the results are limited to one group of stakeholders and do not include the views of patients, hospitals, and robotic equipment makers. The research questions concern the surgeons' point of view, and the research framework was designed around a few factors with a direct constraint on the scope of this study. These factors include the impact of performing robotic-assisted surgery versus traditional procedures, surgeons' culture, the overall success or failure of robotic-assisted surgery, the familiarity and usage of robotic technology in surgery generally, and the differences in perceptions and opinions between surgeons. This study is limited to interviewing surgeons from Indiana; it is not a representation of all the surgeons in the US or surgeons in other countries that use robotic-assisted surgical techniques. Another limitation of this study is the sample size.

One potential bias in this research study is that all robotic-assisted surgeons who were interviewed were using the da Vinci surgical system. Their experiences do not necessarily represent the experiences of surgeons using other robotic systems or surgeons working outside the US. Another potential bias is one of self-selection: surgeons who agreed to be interviewed may have been particularly interested in this technology. In contrast, the surgeons who declined to be interviewed may have had valid input, but were not sufficiently interested to participate.

The modified UTAUT model requires revalidation in a quantitative follow-up study owing to the addition of the Attitude Toward Using Technology and Leadership constructs. It is important to determine whether these constructs have validity, including incremental validity, in the context of robotic-assisted surgery.

### Contextualized Solutions

This qualitative research has adapted the UTAUT model and contextualized it for robotic-assisted surgery. Extending the research entails further quantitative approaches to measure the detailed impact of each construct in the adoption process; however, based on the results of this study, the major UTAUT constructs affecting both users and nonusers are highlighted and thus various solutions can be contextualized to promote the facilitators and remove the barriers. For example, Perceived Usefulness has been expressed as both a main facilitator and barrier to the adoption of robotic-assisted surgery among both users and nonusers. The detailed comments reveal that the surgeons fall in one of three categories regarding evidence supporting adoption: they are seeking the best medical evidence supporting the usefulness of robotic-assisted surgery; they are unaware of published supporting evidence; or they do distrust the evidence. Validating available outcome-based evidence, disseminating it among potential users, and conducting new clinical trials may alleviate or confirm concerns about robotic-assisted surgery.

### Conclusion

Robotic technology will play an increasingly important role in surgery [3]. This study identifies factors influencing surgeons' perception and acceptance of robotic technology to contextualize the UTAUT model for this domain. The results will help not only healthcare institutions and medical technology companies but also medical schools to understand the facilitators and barriers to the acceptance and use of robots in surgical procedures. With this understanding, healthcare institutions and medical technology companies can develop strategic plans and incentives to persuade surgeons to employ robotic-assisted surgery in their routine practice, and medical schools can introduce appropriate training programs.

The results show that most surgeons were attracted to the benefits provided to their patients by performing surgery robotically. Another important factor was the surgeons' attitude: Robot users were more open to change and enjoy the adventure of learning new technologies. Even though they acknowledged the steep learning curve for robotic-assisted surgery, they recognized the potential advantages. Several barriers recognized by the nonusers are the steep learning curve, the lack of incentives or encouragement from the hospitals, and the high cost of the robot. For surgeons to adopt robotic technology, these issues must be addressed. To overcome the lack of tactile feedback, hospitals should provide more opportunities for surgeons to practice using robotic techniques. Robot manufacturers should also investigate haptic technologies for providing tactile feedback.

In Indiana only 14% of hospitals have surgical robots, though they are more prevalent in the greater Indianapolis area. Their high cost is still a barrier for rural hospitals. In Indianapolis, surgeons have more opportunities to gain experience with surgical robots by scrubbing in with a practiced surgeon. Once they can perform robotic-assisted surgery on their own, their patient population is likely to increase in practices lacking robotic surgeons.

Contextualization of the UTAUT model resulted in the merger of some of the subconstructs and the addition of two new main constructs: Attitude toward Using Technology and Leadership. The new modified UTAUT model will enable future research to further enhance the application of technology acceptance models to robotic-assisted surgery. Further quantitative measures, such as scaled questionnaires, can be created based on the modified UTAUT model to measure the prediction rate of each UTAUT construct in the adoption of robotic-assisted surgery. This has practical implications, because it will enable researchers, healthcare managers, and policy makers to measure the readiness of their surgeons in adopting robotic technology.

We believe that conducting this elicitation study with surgeons will lead to more refined, contextualized theories of robotic-assisted surgery acceptance and use. This development is similar to previous studies in other industries [36]. Indeed, in this study we learned not only the salient beliefs of surgeons but also the reasons for those beliefs.

## Methods

To understand surgeons' perspectives on robotic-assisted surgical technology, this study's data was collected through semi-structured interviews. This qualitative method was selected, because little prior research has been conducted on surgeons' adoption of robotic surgical systems and, therefore, the most relevant issues have not yet been identified. Semi-structured interviews gather detailed information about each surgeon's beliefs and behaviors without preconceived factors; the method provides opportunities for identifying unanticipated outcomes and is effective in understanding attitudinal and behavioral nuances in the situated context [37]. Hence, the results of this qualitative study will enable the development of theories and the framing of hypotheses in future quantitative or mixed-methods studies.

### Ethics Statement

This study was approved on May 28, 2008 by the IUPUI/Clarian Research Compliance Administration. The study approval ID is EX0805-47. Informed consent was obtained in writing from all participants immediately before the interviews commenced. The content of the consent form was approved by the above Institutional Review Board. The signed consent form of each participant will be retained for three years. All stored data is deidentified.

### Participants

The sample population was comprised of surgeons in Indiana who practice in robotic-assisted surgery subspecialties. A sample of surgeons was recruited by telephone from a list provided by the Indiana State Medical Association. The goal was to recruit approximately 20 surgeons with a roughly equal number of users and nonusers in each subspecialty. Sixty-eight surgeons were contacted. Among the surgeons agreeing to interviews, 21 were selected and interviewed between June 18 and August 21, 2008 at their offices. Ten of the surgeons were using the da Vinci robot, and the remaining 11 were not. The participating surgeons were from the following specialties: urological surgery, cardiovascular surgery, cardiothoracic surgery, obstetric and gynecological (OB/GYN) surgery, and general surgery.

In this study's sample, 4 of 7 OB/GYN and 4 of 7 urology surgeons were robot users. By contrast, only 1 of 4 surgeons in the cardiovascular specialty and 1 of 3 in general surgery were robot users. Among the robot users, there was considerable variation in the number of robotic surgeries performed annually. For example, one surgeon used robotic technology in 100% of surgical cases, averaging 200 to 250 cases per year, whereas another surgeon used robotic technology in less than 2% of surgical cases (just 4 of 150 to 200 cases per year). On average the users had performed robotic-assisted surgery for 3.7 years (*SD* = 1.9 years). The largest age group among users was 40–49 years (50%). Surprisingly, the largest age group among nonusers was younger: 30–39 years (45%). Eighteen of the 21 participants (86%) were male: Nine of 10 users (90%) and 9 of 11 nonusers (82%).

### Data Collection Instruments and Analysis Procedures

Content analysis was used in conjunction with the UTAUT model to analyze the conversations between the researcher and participating surgeons. Content analysis is the systematic, objective, and quantitative analysis of message characteristics by inductive methods [38]. Thus, content analysis was used to identify new constructs to augment the UTAUT model and subconstructs that can be combined in the context of robotic-assisted surgery.

This study used Kvale's [39] approach for data analysis, which has seven steps: thematizing, designing, interviewing, transcribing, analyzing, verifying, and reporting. Given the theme of technology acceptance in robotic-assisted surgery, semi-structured interviews were designed before being conducted with the surgeons (Appendix S1). A demographics questionnaire was administered before the interviews (Appendix S2). During the interviews audio was recorded. Upon completion of the interviews, collected data were analyzed. Interviews were transcribed and processed by the principal researcher and an independent researcher. During processing, pronouns, and other indexical terms were replaced with their nominal meanings, so that their surrounding concepts would be clear even when separated from the immediate context of the transcription. Each transcription was then divided and reorganized into groups of concept. The concepts were then separated into those from robot users and nonusers. For each of these participant groups, the concepts, hereafter referred to as *comments*, were categorized according to the UTAUT model.

To help contextualize the UTAUT model for robotic-assisted surgery, the following modifications were proposed (Figure 1):

#### Model Streamlining

Each main construct of the UTAUT model includes two or more subconstructs. However, some of the subconstructs have similar definitions in the context of robotic-assisted surgery. Because the same comment could often be categorized under multiple subconstructs, streamlining these subconstructs was necessary after the interviews. Hence, similar subconstructs were combined, such as the Perceived Ease of Use, Complexity, and Ease of Use subconstructs of Effort Expectancy. Another issue with UTAUT is that some of the subconstructs constitute the positive and negative anchors of the same dimension, while other subconstructs have no opposing anchors. This deficiency had to be remedied so that we could place pro-adoption and contra-adoption comments under each subconstruct.

#### Main Construct Addition

The UTAUT constructs are significant in determining users' behavioral intention and actual use of a technology. In this study, almost half the participants had already adopted robotic technology; some of them were even involved in promoting the technology to their colleagues and assisting robot developers in improving it. When the comments were categorized, some comments did not fit into existing UTAUT constructs. Because many of these comments reflected the surgeons' technology adoption behavior, two new constructs were added, Attitude toward Using Technology and Leadership, thus expanding the UTAUT model to six constructs. In the validation of the model, Venkatesh et al. [26] found the observed relation between Attitude Toward Using Technology and Behavioral Intention to be spurious because both constructs are strongly affected by Performance Expectancy and Effort Expectancy. However, in this qualitative study, the comments on Attitude Toward Using Technology were valuable regardless of their incremental validity. Figure 1 depicts the expanded UTAUT model developed by the aforementioned contextualization methods.

The following methods were used to assess and enhance reliability and validity:

#### Reliability

When using human coding, intercoder reliability must be established. Intercoder reliability is the number of times two coders agreed on a unit divided by the total number of units coded [38]. Two coders working separately divided the transcriptions of the interviews into concepts, categorized these comments, and compared the results. The initial intercoder reliability was 74%. Disagreements in coding were discussed until a consensus was achieved.

#### Validity

Two validation procedures were employed in this research study: (1) participant data triangulation—facts and opinions provided by one participant were checked by other participants to build a coherent justification for the choice of constructs; and (2) member-checking—each participant evaluated the accuracy of his or her categorized comments. Five participants proposed minor corrections to their comments, which were then incorporated into the results.

## Supporting Information

Appendix S1 Interview Guiding Framework
(DOC)

Appendix S2 Demographics Questionnaire
(DOC)

## References

[1] Bergeron B (2002). Achieving clinician buy-in to technology. Medscape General Medicine 4(4).. http://www.medscape.com/viewarticle/446224.

[2] Talamini MA, Hanly EJ (2005). Technology in the operating suite.. Journal of the American Medical Association.

[3] Morris B (2005). Robotic surgery: Applications, limitations, and impact on surgical education.. Medscape General Medicine.

[4] Holden RJ, Karsh TB (2009). Atheoretical model of health information technology usage behaviour with implications for patient safety.. Behaviour & Information Technology.

[5] Haggag AA (2006). Robotic surgery: When technology meets surgical precision.. Internet Journal of Health.

[6] Tabor W (2007). On the cutting edge of robotic surgery.. Nursing.

[7] Furukawa T, Morikawa Y, Ozawa S, Wakabayashi G, Kitajima M (2001). The revolution of computer-aided surgery: The dawn of robotic surgery.. Minimal Invasive Theraphy & Allied Technology.

[8] Karamanoukian RL, Donias HW, Glick PL, Bergsland J, Karamanoukian H (2002). Survey of resident training in robotic surgery.. American Surgeon.

[9] Gomez G, Townsend CM, Beauchamp RD, Evers BM, Mattox KL (2004). Emerging technology in surgery: Informatics, electronics, robotics.. Sabiston Textbook of Surgery, 17th ed.

[10] Meadows M (2002). Robots lend a helping hand to surgeons.. FDA Consumer.

[11] Lanfranco AR, Castellanos AE, Desai JP, Meyers WC (2004). Robotic surgery: A current perspective.. Annals of Surgery.

[12] Lowenfels AB (2004). Robotics.. http://www.medscape.com/viewarticle/511854_4.

[13] Nguyen NT, Hinojosa MW, Finley D, Stevens M, Paya M (2004). Application of robotics in general surgery: Initial experience.. American Surgeon.

[14] Finan MA, Rocconi RP (2010). Overcoming technical challenges with robotic surgery in gynechologic oncology.. Surgical Endoscopy.

[15] von Gruenigen VE, Sawyer MD, Ponsky LE, Hurd WW (2009). Recent innovations in minimally invasive surgery and implications for gynecology.. Journal of Gynecology Surgery.

[16] Marescaux J, Rubino F (2005). Robotic surgery: Potentials, barriers, and limitations.. European Surgery.

[17] Van J (2007, Sept 7). Robotic help at heart of hospital's initiative - U. of C. hires expert in bypass procedure. Chicago Tribune.. http://www.highbeam.com/doc/1G1-168370175.html.

[18] Mathews CA (2010). Application of robotic surgery in gynecology.. Journal of Woman's Health.

[19] Berci G, Phillips E, Fujita F (2004). The operating room of the future: What, when and why?. Surgical Endoscopy.

[20] Murphy DA, Miller JS, Langford DA, Snyder AB (2006). Endoscopic robotic mitral valve surgery.. The Journal of Thoracic and Cardiovascular Surgery.

[21] Nazemi T, Galich A, Smith L, Balaji K (2006). Robotic urological surgery in patients with prior abdominal operations is not associated with increased complications.. International Journal of Urology.

[22] Bocca S, Stadtmauer L, Oehninger S (2007). Current status of robotically assisted laparoscopic surgery in reproductive medicine and gynaecology.. Reproductive BioMedicine Online.

[23] McLeod IK, Mair EA, Melder PC (2005). Potential applications of the da Vinci minimally invasive surgical robotic system in otolaryngology,. ENT - Ear, Nose & Throat Journal.

[24] Geffen D (2009). UCLA Center for Advanced Surgical and Interventional Technology.. http://casit.ucla.edu/body.cfm?id=13.

[25] Yarbrough AK, Smith TB (2007). Technology acceptance among physicians: A new take on TAM.. Medical Care Research and Review.

[26] Venkatesh V, Morris MG, Davis GB, Davis FD (2003). User acceptance of information technology: Toward a unified view.. Management Information Systems Quarterly.

[27] Chau PYK, Hu PJ (2002). Examining a model of information technology acceptance by individual professionals: An exploratory study.. Journal of Management Information Systems.

[28] Schurr M, Arezzo A, Buess G (1999). Robotics and systems technology for advanced endoscopic procedures: Experiences in general surgery.. European Journal of Cardiothorac Surgery.

[29] Camarillo D, Krummel T, Salisbury K (2004). Robotic technology in surgery: Past, present, and future.. American Journal of Surgery.

[30] Davis FD (1989). Perceived usefulness, perceived ease of use, and user acceptance of information technology.. MIS Quarterly.

[31] Davis FD, Bagozzi RP, Warshaw PR (1989). User acceptance of computer technology: A comparison of two theoretical models.. Management Science.

[32] Ajzen I, Fishbein M (1980). Understanding attitudes and predicting social behavior..

[33] Fishbein M, Ajzen I (1975). Belief, attitude, intention, and behavior: An introduction to theory and research..

[34] Malhotra Y, Galletta DF (1999). Extending the technology acceptance model to account for social influence: Theoretical bases and empirical validation.. Proceedings of the 32nd Hawaii International Conference on System Sciences.

[35] Rawstorne P, Jayasuriya R, Caputi P (2000). Issues in predicting and explaining usage behaviors with the technology acceptance model and the theory of planned behavior when usage is mandatory.. Proceedings of the 21st International Conference on Information Systems.

[36] Holden RJ, Karsh BT (2010). The technology acceptance model: Its past and its future in health care.. Journal of Biomedical Informatics.

[37] Creswell JW (2003). Research design qualitative, quantitative, and mixed methods approaches, 2nd ed..

[38] Neuendorf KA (2002). The content analysis guidebook..

[39] Kvale S (1996). InterViews: An introduction to qualitative research interviewing..

