# Supplementary material for: Facilitators and Barriers to Adopting Robotic-Assisted Surgery: Contextualizing the Unified Theory of Acceptance and Use of Technology
Source: PLoS One. 2011 Jan 20;6(1):e16395. doi: 10.1371/journal.pone.0016395 (PMC3024425; doi:10.1371/journal.pone.0016395)
Supplement: Appendix S1 — Interview Guiding Framework (DOC) [file pone.0016395.s001.doc]

**Appendix S1: Interview Guiding Framework**

(a) Performance Expectancy and Effort Expectancy

1. Do you perform Robotic Surgery?
2. What made you decide to adopt robotic surgery in your routine practice?
3. What are the benefits of robotic surgery?
4. What are the advantages compared with traditional surgery or MIS?
5. What are the disadvantages of robotic surgery?
6. How comfortable are you performing robotic surgery?
7. How do you apply robotic surgical techniques to enhance your performances and outcomes?
8. How did you come to trust this technology?

(b) Social Influence

1. Have you encouraged your colleagues to adopt this technology?
2. What have you done to encourage them?
3. How enthusiastically have you advocated the use of robotic surgical techniques?
4. What kind of affect personally to you since you started performing robotic surgery?
5. How many surgeons in your practice/organization are currently using robotic assisted techniques?
6. Have you observed a robotic surgical procedure? What do you think about it?
7. Since your organization has already adopted this technology, why have you not adopted this technology in your routine practice?

(c) Facilitating Conditions

1. What type of training did you receive before you started to perform robotic surgeries?
2. How long is the training?
3. What do you think the training program should be?
4. What type of continuing education program do you need to maintain your current license or certificate in open surgery or MIS or robotic surgery?
5. What kind of technical support do you receive?
6. How do you feel about the support you are receiving from management?
7. What does management in your organization do to encourage you to perform robotic surgery?
8. Are you satisfied with the kind of support you have received?
9. What kind of incentives do you receive from your organization for performing robotic surgery?
10. What do you tell your patient about robotic surgery when asked?

(d) Attitude toward Use

1. What do you expect from robotic surgery?
2. What would you expect from this technology before you would be willing to adopt it in your practice?

(e) Leadership

1. Were you involved in the decision of your organization to adopt this technology?
2. How involved were you?
