# Supplementary material for: Facilitators and Barriers to Adopting Robotic-Assisted Surgery: Contextualizing the Unified Theory of Acceptance and Use of Technology
Source: PLoS One. 2011 Jan 20;6(1):e16395. doi: 10.1371/journal.pone.0016395 (PMC3024425; doi:10.1371/journal.pone.0016395)
Supplement: Appendix S2 — Demographics Questionnaire (DOC) [file pone.0016395.s002.doc]

**Appendix S2: Demographics Questionnaire**

1. Age (obtain a range)
   1. 30 – 40
   2. 41 – 50
   3. 51 – 60
   4. > 60
2. Gender (observation)
   1. Male
   2. Female
3. Specialty (can gather prior to interview)
   1. Cardiovascular
   2. Urology
   3. OB/GYN
   4. General Surgery
   5. Neurosurgery
4. How many years have you been in practice? (Possibly obtain this information prior to the interview)
5. How many surgeries do you perform each year? (possibility obtain this information from the office)
6. How many years ago did you perform your first robotic surgery?
7. How comfortable are you with computers?
8. How comfortable are you with computer games?
9. How comfortable are you with the rapid change in technology in the medical field?
   1. List of procedures that can be performed with robots: (a) Prostatectomy (b) Gastric Bypass (c) Hysterectomy (d) Myomectomy (e) Sacrocolpopexy (f) Esophagectomy (g) Mitral Valve Repair/replacement (h) Thymectomy (i) Lobectomy (j) Mediastinal Tumor Resection (k) Pyelopolasty (l) Cystectomy (m) Nephrectomy (n) Ureteral Reimplantation (o) Colorectal Surgery (p) Nissen Fundoplication
